# Risk factors and clinical characteristics for bronchopulmonary dysplasia associated pulmonary hypertension in very-low-birth-weight infants

**DOI:** 10.1186/s12872-021-02330-w

**Published:** 2021-10-24

**Authors:** Junfang Sun, Bowen Weng, Xiaoyue Zhang, Xiaoyun Chu, Cheng Cai

**Affiliations:** 1 Department of Respiratory Medicine, Shanghai Children’s Hospital, Shanghai Jiao Tong University, Shanghai, China; 2 Department of Neonatology, Shanghai Children’s Hospital, Shanghai Jiao Tong University, Shanghai, 200062 China

**Keywords:** Risk factor, Bronchopulmonary dysplasia, Very-low-birth-weight infants, Pulmonary hypertension

## Abstract

**Background:**

Pulmonary hypertension (PH) is a common complication of bronchopulmonary dysplasia (BPD) in very-low-birth-weight infants (VLBWIs). Although recent studies have increased awareness that PH contributes significantly to the high morbidity and mortality of BPD, the risk factors and clinical characteristics for PH in VLBWIs are little known.

**Objectives:**

To investigate the risk factors and clinical characteristics for BPD-associated pulmonary hypertension (BPD-PH) in VLBWIs.

**Methods:**

A retrospective case–control observational study of VLBWIs with BPD admitted to a neonatal intensive care unit (NICU) over 4 years. According to echocardiograms confirming elevated pulmonary artery pressure after 28 days after birth, we divided BPD infants into PH group (n = 18) and non-PH group (n = 65). We compared pre- and postnatal characteristics between VLBWIs with or without PH. Multivariable logistic regression analysis was conducted with backward selection.

**Results:**

A total of 83 infants with BPD were divided into PH group (n = 18) or non-PH group (n = 65). The average birth weight of the infants with BPD was 1078.1 g. Compared with those infants of the non-PH group, the birth weight of BPD-PH infants was significantly lower (968.1 ± 187.7 vs. 1108.5 ± 185.8,* P* = 0.006). Infants in the PH group had a higher incidence of patent ductus arteriosus (PDA) and underwent longer durations of oxygen therapy and mechanical ventilation compared to those in the non-PH group. In all subjects, birth weight (OR 0.995; 95% CI 0.991–0.999; *P* = 0.025) and PDA (OR 13.355; 95% CI 2.950–60.469; *P* = 0.001) were found to be specific risk factors for BPD-PH in this cohort.

**Conclusions:**

The study shows PDA and birth weight are specific risk factors for BPD-PH in VLBWIs.

## Background

Recently, owing to the use of prenatal steroids and postnatal surfactant (PS), improved ventilator strategies and advanced nursing techniques, the survival rate of premature infants especially VLBWIs and extremely low birth weight infants (ELBWIs) have increased [1]. In addition, BPD as a common chronic pulmonary complication of premature infants, has differed from its classical form described by Northway in 1967 [2–4]. Compared with the classical BPD, there are histologic differences in the “new” BPD, including simpler structure and reductions in the number of alveoli [5].

VLBWIs with BPD usually have a high risk of cardiovascular sequelae [6]. Among the cardiovascular sequelae, PH is associated with high morbidity and mortality in premature infants with BPD. Vascular remodeling, reduced alveolar–capillary surface area, abnormal vascular tone and reactivity lead to an increase in pulmonary vascular resistance (PVR) [7]. Increased PVR results in increased pulmonary arterial pressure.

At present, few studies on the clinical characteristics and outcomes of PH in VLBWIs with BPD have been published. Little is known about the risk factors for PH in VLBWIs with BPD, making it difficult to formulate screening strategies to identify PH in these infants. The aim of this study was to determine the clinical characteristics of PH associated with BPD in VLBWIs and investigate the risk factors for the disease.

## Material and methods

### Study design

This was a retrospective study, conducted by reviewing medical records and data. The study protocol was approved by the institutional research ethics committee of Shanghai Children’s Hospital (2015RY009-F01).

### Patients

**Fig. 1:**
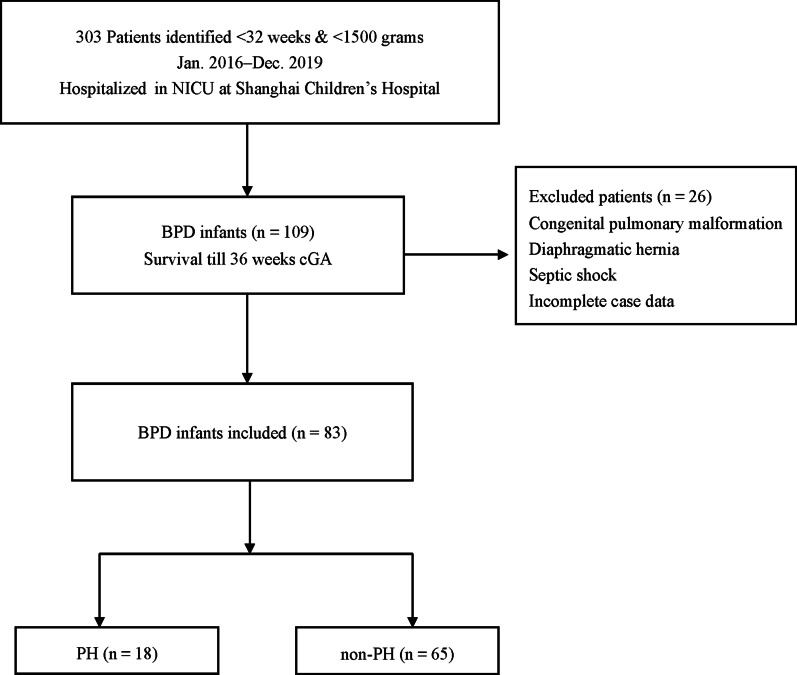
Flow diagram showing the study design of BPD-PH infants enrolled in this study

The retrospective review was performed of data from 109 infants with a gestational age of < 32 weeks and a birth weight < 1500 g who were hospitalized between January 2016 and December 2019 in the NICU at Shanghai Children’s Hospital. The exclusion criteria were congenital pulmonary malformation, diaphragmatic hernia, septic shock or incomplete case data (n = 26). None of the twenty-six excluded cases have undergone echocardiography screening at the above time. The case data was incomplete. Infants with a diagnosis of BPD [8] were divided into two groups, PH group and non-PH group, according to result of the echocardiogram screen conducted after at least 28 days after birth (over 36 weeks’ corrected gestation or before discharged home). After recognition of PH, 2 infants passed away in the PH group and 1 in the non-PH group (11% vs. 2%). Collect and retrospectively analyze data on the clinical characteristics of patients. The flow diagram showing the study design of the 83 premature infants with BPD and PH enrolled in this study is presented in Fig. 1.

### Definitions

#### Bronchopulmonary dysplasia

We use the consensus definition of BPD for infants with GA < 32 weeks to classify our patient population [8]. Currently, the definition of BPD is based on respiratory support and supplemental oxygen therapy that a patient’s need for at 36 weeks corrected gestational age (cGA). The diagnostic criteria for BPD in this study are based on the definition proposed in the conference of the National Institute of Child Health and Human Development (NICHD) in June 2000, infants suffer from BPD with supplemental oxygen required for a minimum of 28 days [8]. The definition, which categorizes the severity of BPD, is proposed (Table 1).

**Table 1:** NICHD diagnostic criteria for severity of BPD

BPD severity	Criteria
Mild	Supplemental oxygen required ≥ 28 days, termination of supplemental oxygen by 36 weeks cGA or discharge
Moderate	Supplemental oxygen required ≥ 28 days, requirement of < 30% O_2_ at 36 weeks cGA or discharge
Severe	Supplemental oxygen required ≥ 28 days, requirement of ≥ 30% O_2_ and/or continuous positive airway pressure or mechanical ventilation at 36 weeks cGA or discharge

#### Pulmonary hypertension

In the present study, the diagnosis of PH depended entirely on echocardiography. The echocardiography was performed after at least 28 days after birth (over 36 weeks’ corrected gestation or before discharge to home) for PH [9]. All echocardiographic examinations were conducted by a certain pediatric cardiologist team. The most objective measure of PH by echocardiogram is the estimated right ventricular systolic pressure (RVSP) derived from the tricuspid regurgitant jet velocity. The calculation of a systolic RV-to-right atrium pressure gradient by using the simplified Bernoulli equation (pressure gradient = 4 × jet velocity^2^). The criteria for PH were met by any of the following findings [10, 11]: (1) RVSP greater than 40 mmHg; (2) RVSP/systemic systolic blood pressure greater than 0.5; (3) Cardiac shunt with bidirectional or right-to-left flow; (4) If no tricuspid regurgitation (TR) shunt presents then two out of following three criteria: (a) Any degree of interventricular septal flattening; (b) Right ventricular dilatation; (c) Right ventricular hypertrophy.

#### Clinical variables

The clinical characteristics were directly evaluated by a certain reviewer from medical records. Data extracted from the patient’s medical records included birth history, maternal medical history, prenatal characteristics, postpartum medical history, and various complications. Prenatal diagnosis, such as gestational diabetes, gestational hypertension, preterm premature rupture of membranes (PPROM) is defined in accordance with the American College of Obstetrics and Gynecology (ACOG) guidelines [12–14]. Placental abruption is classically defined as the complete or partial separation of a normally implanted placenta before delivery [15]. Modified Bell’s criteria were used to classify necrotizing enterocolitis (NEC) [16]. Hypoxemia was defined as arterial partial pressure of oxygen less than 60 mm of mercury. This value was extracted from the results of arterial blood gas analysis after the diagnosis of BPD and before the detection of PH by echocardiography.

### Statistical analysis

All statistical analyses were performed using SPSS for Windows, version 27.0 Data were presented as mean ± SD or frequency. Chi-square test was used to analyze categorical variables. Independent sample t-test was used to compare normally distributed continuous variables. Risk factors for BPD-PH were analyzed with binary logistic regression model. The variables included in the multivariate analyses for BPD-PH were gestational age, birth weight, PDA, duration of O_2_ therapy and duration of mechanical ventilation. A stepwise regression was utilized and in all analyses. *P* < 0.05 was considered significant.

## Results

### Patient selection

As per Fig. 1, we found 109 preterm infants with BPD, whose birth weight (BW) was less than 1500 g. According to the exclusion criteria, we had excluded 26 patients. The excluded cases included 2 severe BPD infants, 4 moderate BPD infants, and 20 mild BPD infants. A total of 83 infants with BPD were divided into PH group (n = 18) or non-PH group (n = 65).

### Birth history

Compared with those premature infants of the non-PH group, the birth weight of BPD-PH infants was significantly lower (*P* = 0.006). But there were no significant differences in sex, gestational age, small for gestational age (SGA) and Apgar score after five minutes (*P* > 0.05) (Table 2).

**Table 2:** Baseline clinical characteristics of the study population

Clinical characteristics	All study patients (n = 83)	Non-PH (n = 65)	PH (n = 18)	*P*
*Birth history*				
Male	46(55%)	35(54%)	11(61%)	0.583
Birth weight, g	1078.1 ± 194.0	1108.5 ± 185.8	968.1 ± 187.7	0.006
Gestational age, weeks	28.3 ± 1.3	28.4 ± 1.2	27.9 ± 1.3	0.132
*5-min Apgar scores*				
0–3	1(1%)	0(0)	1(6%)	
4–7	15(18%)	13(20%)	2(11%)	0.120
8–10	67(81%)	52(80%)	15(83%)	
Small for gestational age	5(6%)	2(3%)	3(17%)	0.066
*Maternal medical history*				
Cesarean section	41(49%)	32(49%)	9(50%)	0.954
Maternal age, ≥ 35	14(17%)	9(14%)	5(28%)	0.162
Gestational diabetes	7(8%)	5(8%)	2(11%)	0.644
Gestational hypertension	14(17%)	11(17%)	3(17%)	0.979
PPROM	18(22%)	14(22%)	4(22%)	0.950
Placental abruption	11(13%)	9(14%)	2(11%)	0.762
*Clinical characteristics*				
BPD severity				
mild	38(46%)	32(49%)	6(33%)	
moderate	27(32%)	22(34%)	5(28%)	0.130
severe	18(22%)	11(17%)	7(39%)	
Pulmonary surfactant	73(88%)	56(86%)	17(94%)	0.339
Respiratory distress syndrome	82(99%)	64(98%)	18(100%)	0.597
Duration of O_2_ therapy, days	58.9 ± 28.0	55.5 ± 27.8	70.9 ± 26.1	0.038
Duration of mechanical ventilation, days	51.4 ± 27.5	48.0 ± 27.2	63.9 ± 25.4	0.028
Duration of hospitalization, days	75.0 ± 24.5	73.1 ± 25.2	81.9 ± 20.8	0.178
Hypoxemia	81(98%)	63(97%)	18(100%)	0.451
NEC, stage ≥ 2	6(7%)	5(8%)	1(6%)	1.000
Home oxygen	3(4%)	2(3%)	1(6%)	0.525
Death	3(4%)	1(2%)	2(11%)	0.117
*Transthoracic echocardiography*				
Patent ductus arteriosus	36(43%)	21(32%)	15(83%)	0.001
Atrial septal defect	66(80%)	51(78%)	15(83%)	0.650
Patent foramen ovale	34(41%)	29(45%)	5(28%)	0.199
Ventricular septal defect	1(1%)	1(2%)	0(0)	0.597

### Maternal medical history

There were no significant differences between the patients of the two groups in terms of maternal age, cesarean section, gestational hypertension, gestational diabetes, placental abruption, and preterm premature rupture of membranes (*P* > 0.05) (Table 2).

### Clinical characteristics

Compared with infants in the non-PH group, the proportion of moderate to severe BPD infants in the PH group did not increase significantly (*P* = 0.130) (Table 2). Compared with infants without PH, infants with PH had a greater need for longer durations of oxygen therapy (*P* = 0.038) and mechanical ventilation (including invasive ventilation and non-invasive ventilation) (*P* = 0.028), as shown in Table 2. The proportion of hypoxemia did not differ between infants with and without PH (*P* = 0.451) (Table 2). After recognition of PH, 2 patients passed away in the PH group and 1 in the non-PH group (11% vs. 2%). There was no significant difference in mortality (*P* = 0.117) (Table 2).

### Transthoracic echocardiography

Compared with those premature infants in the non-PH group, the incidence of PDA was significantly higher in the PH group (*P* = 0.001) (Table 2). In the study, 4 patients underwent ligation of PDA, all of them in the PH group. Twenty-two patients were treated with NSAIDs to close the ductus, and 9 of them were in PH group. Compared with that of the non-PH group, there were no significant differences in other echocardiography results of the PH group (*P* > 0.05) (Table 2).

### Logistic regression

Univariate analysis for the comparison of clinical characteristics according to the presence or absence of PH in all subjects included in this study showed a statistically significant difference (*P* < 0.05) in five variables (Table 2): birth weight (968.1 ± 187.7 vs. 1108.5 ± 185.8, *P* = 0.006), duration of oxygen therapy (70.9 ± 26.1 vs. 55.5 ± 27.8, *P* = 0.038), duration of mechanical ventilation (63.9 ± 25.4 vs. 48.0 ± 27.2, *P* = 0.028), and PDA (83% vs. 32%, *P* = 0.001). Based on the P values on univariate analyses, a multivariate logistic regression model was performed to ascertain risk factors for development of BPD-PH. In all subjects, birth weight (OR 0.995; 95% CI 0.991–0.999; *P* = 0.025) and PDA (OR 13.355; 95% CI 2.950–60.469; *P* = 0.001) were found to be specific risk factors for BPD-PH in this cohort (Table 3).

**Table 3:** Risk factors for BPD-PH in VLBWIs by binary logistic regression analysis

Clinical characteristics	OR	95%CI	*P*
Birth weight, g	0.995	0.991–0.999	0.025
Gestational age, weeks	1.414	0.761–2.628	0.273
Duration of O_2_ therapy, days	0.988	0.937–1.042	0.663
Duration of mechanical ventilation, days	1.021	0.964–1.081	0.472
Patent ductus arteriosus	13.355	2.950–60.469	0.001

## Discussion

This retrospective study sought to determine the risk factors and clinical characteristics of infants with BPD-PH. In recent years, the incidence of BPD is on the rise because of the advances in NICU [17]. Prior studies have reported that the incidence of BPD-PH ranged from 8 to 36% [18]. This broad range may be explained by the heterogeneity of the studied populations, by varying management practices, or by the variability in the definition of PH. In this study, the incidence of PH in premature infants with BPD was 21.7% (18/83). This incidence in the cohort was close to previous reports.

The causation is multifactorial in BPD-PH. We confirmed several well-known factors [19], and found the factors were also present in VLBW premature patients with BPD-PH. As previously shown, several factors associated with development of PH in BPD patients, including BW, small for gestational age, PDA, duration of oxygen therapy and duration of mechanical ventilation [20–23]. In the present study low birth weight was associated with the development of PH. However, we did not identify these SGA as risk factor for BPD-PH in the present study. The developmental stage of the lung tissue of infants with moderate to severe BPD was from the tubule stage to the vesicle stage. The pulmonary vascular abnormalities in BPD including impaired pulmonary angiogenesis, abnormal pulmonary vascular remodeling, heightened pulmonary vascular tone, and development of abnormal collateral circulations [4]. Several studies have shown that about 25% of premature infants with moderate to severe BPD would develop BPD-PH [10, 24]. On the contrary, this study did not find that the severity of BPD was related to the development of PH. In the study, the duration of medical ventilation and oxygen therapy was significantly longer in the PH group than in the non-PH group. These findings are consistent with previous finding by Nagiub et al. [20]. It was also emphasized from the Arjaans and coworkers’ study, that prolonged invasive mechanical ventilation would aggravate lung inflammation, causing pulmonary vascular remodeling, disrupting the normal lung development of premature infants [25]. Besides, the results of univariate analysis of this study found that the birth weight in PH group was significantly less than that in the non-PH group. A recent study by Collaco and coworkers described the risk factors for BPD-PH including low birth weight, low gestational age, and prolonged oxygen therapy [26]. Immature lung development is the root cause of BPD. Respiratory support is one of the important treatment measures for BPD. Prolonged oxygen therapy in PH group is expected. However, due to long-term exposure to the ventilator, the immature lung tissues will experience pulmonary inflammation, capillary endothelial cells damage and vascular remodeling, leading to increased pulmonary artery pressure. In this study, all patients with BPD received nebulized budesonide for addressing pulmonary inflammation. Therefore, we should adopt protective ventilation strategies to minimize the damage caused by mechanical ventilation [27]. Among 18 patients with PH, only 1 was treated with sildenafil. There was no significant difference in mortality between the two groups (*P* = 0.117). A number of reasons could explain the lack of significant association with mortality, among them, the most likely one is probably the difference in our patient/referral population.

Abnormal pulmonary circulation in patients with BPD, such as persistent ductus arteriosus, was related to the occurrence of PH. The pulmonary blood flow increased due to ductal shunting. The increased blood flow through an immature pulmonary bed produces vascular remodeling that results in a postnatal increase in pulmonary vascular resistance [28]. In the present study, the incidence of PDA in the PH group was significantly higher than that in the non-PH group, which is consistent with previous reports. In addition, 4 patients underwent ligation of PDA, all of them in the PH group. Twenty-two patients were treated with NSAIDs to close the ductus, and 9 of them were in PH group. Bancalari and coworkers found that the persistence of PDA in BPD infants leads to continuous body-pulmonary shunt, which may be related to increased pulmonary artery pressure [28]. However, the PDA may also be the consequence of PH. Patients with PH may keep their ductus arteriosus patent to reduce right ventricular afterload, as a response mechanism. The interaction between PDA and PH needs further research.

## Conclusion

In conclusion, we found that BW, PDA, duration of oxygen therapy and duration of mechanical ventilation were risk factors for BPD-PH. The survival rate of ones with PH was lower than that of infants who did not. Furthermore, the infants with PH had to undergo longer durations of oxygen therapy and mechanical ventilation than those without PH. PDA was a specific risk factor for PH in VLBWIs with BPD. Therefore, we suggest that a more active screening echocardiogram may be needed for diagnosing PH in VLBWIs with BPD. Further large cohort studies will be required to identify other risk factors for the development of PH in VLBWIs with BPD.

### Limitations

This study has several limitations, including the lack of confirmation of PH by right heart catheterizations, which is the gold standard for diagnosis, because performing the invasive evaluations was not practical in this population. In addition, the study was a single-center retrospective clinical study and we lack the data on pre-natal steroid exposure. Besides, that measurements of these cardiac ultrasound results were not repeated from the raw images, but rather collected from individual clinical reports.

## Data Availability

The datasets used and/or analyzed during the study is available from the corresponding author on reasonable request.

## Abbreviations

PH: Pulmonary hypertension
BPD: Bronchopulmonary dysplasia
VLBWIs: Very-low-birth-weight infants
BPD-PH: BPD-associated pulmonary hypertension
NICU: Neonatal intensive care unit
PDA: Patent ductus arteriosus
PS: Postnatal surfactant
ELBWIs: Extremely low birth weight infants
PVR: Pulmonary vascular resistance
cGA: Corrected gestational age
NICHD: National Institute of Child Health and Human Development
RVSP: Right ventricular systolic pressure
PPROM: Preterm premature rupture of membranes
ACOG: American College of Obstetrics and Gynecology
BW: Birth weight
NEC: Necrotizing enterocolitis
SGA: Small for gestational age
